# Interaction of Methanol over CsCl- and KCl-Doped η-Alumina and the Attenuation of Dimethyl Ether Formation

**DOI:** 10.1021/acs.jpcc.2c02275

**Published:** 2022-06-16

**Authors:** Alastair R. McInroy, John M. Winfield, Christopher C. Dudman, Peter Jones, David Lennon

**Affiliations:** † School of Chemistry, University of Glasgow, Joseph Black Building, Glasgow G12 8QQ, U.K.; ‡ Inovyn, South Parade, Runcorn, Cheshire WA7 4JE, U.K.

## Abstract

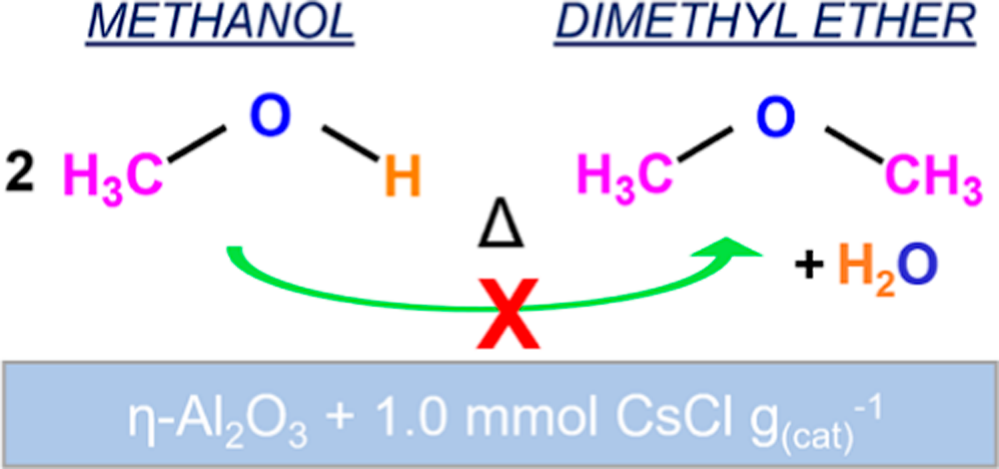

As part of a program to investigate aspects of surface chemistry relevant to methyl chloride synthesis catalysis, the interaction of methanol with η-alumina doped with either CsCl or KCl in the range 0.01–1.0 mmol g_(cat)_^–1^ is investigated by a combination of diffuse reflectance infrared Fourier transform spectroscopy and temperature-programed desorption (TPD). Infrared spectra (IR) recorded at 293 K show that increasing the concentration of the group 1 metal chloride progressively decreases the surface concentration of associatively chemisorbed methanol and changes the environment in which the adsorbed methanol resides. For CsCl concentrations of ≥0.6 mmol g_(cat)_^–1^, chemisorbed methoxy species dominate the IR spectrum, while TPD studies show that the amount of methanol adsorbed onto the surface, and subsequently desorbed unchanged, changes relatively little. In the TPD experiments, some of the adsorbed methanol reacts to give dimethyl ether (DME) which then desorbs; for dopant concentrations of 1.0 mmol g_(cat)_^–1^, DME formation is suppressed to below the limit of detection. Unexpectedly, the presence of formate species generated at 293 K is also observed spectroscopically, characterized by a ν_asym_(COO) mode which exhibits a hypsochromic shift relative to potassium formate; surface concentrations of formate are higher at higher loadings of group 1 metal chloride. Temperature-programed IR spectroscopy shows that the room-temperature formate species desorbs, decomposes, or migrates on warming to 653 K. Thermal ramping of the methanol-saturated surface also results in formate production but one that exhibits an IR profile in agreement with earlier observations and literature values. Increasing the concentrations of the group 1 metal chloride progressively decreases the presence of the thermally induced formate moiety. The study not only reinforces the concept of group 1 metal chloride additives progressively rendering ineffective those Lewis acid sites present at the η-alumina surface which convey discrete reaction characteristics [*e.g.*, (i) dimerization of methanol to form DME and (ii) an activated methoxy → formate transition] but also suggests the generation of reactive sites not present in the undoped alumina.

## 1 Introduction

Methyl chloride synthesis is a reaction of some significance within the chloro-alkali industry.^1^ On the industrial scale, methyl chloride synthesis *via* the heterogeneously catalyzed hydrochlorination of methanol predominates, utilizing catalysts such as Al_2_O_3_-supported ZnCl_2_, CuCl_2_, or H_3_PO_4_^1^
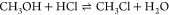
1
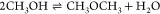
2Equation 1 describes methyl chloride synthesis from the reaction between methanol and anhydrous hydrogen chloride, with the reaction catalyzed at Lewis acid sites.^1^ Selectivity to methyl chloride is reduced by the forward reaction shown in eq 2, where the alcohol is converted to dimethyl ether (DME). An improved methyl chloride synthesis catalyst will catalyze reaction 1 more effectively than reaction 2.^2^

This article examines aspects of the surface chemistry of the methyl chloride synthesis process over η-alumina-based catalysts in the absence of HCl. Specifically, the interaction of methanol on η-alumina that has been modified by the addition of either CsCl or KCl and then activated at 623 K is investigated, with the focus being on retardation of DME formation (eq 2). Earlier infrared (IR) spectroscopic studies utilized pyridine as a probe molecule to discern the acid site distribution of the η-alumina substrate.^3^ Subsequent studies used the same probe molecule to evaluate how the group 1 metal chlorides were able to neutralize selectively Lewis acid sites of the η-alumina to effect site-selective chemistry that is associated with attenuated DME production (eq 2).^4^ The present work concentrates on using methanol as a spectroscopic probe molecule to discern how the group 1 metal chloride may be perturbing the surface chemistry of the reagent. Thus, the work does not directly investigate aspects of methyl chloride synthesis catalysis; rather, it represents a fundamental study of methanol adsorption on catalytically relevant surfaces. For η-alumina/CH_3_OH, temperature-programed IR spectroscopy showed that heating chemisorbed methoxy groups to ≥573 K induced the formation of a bidentate formate species.^5^ Unexpectedly, this investigation shows a modified formate species to be present in a different surface environment at room temperature for heavily doped materials (*e.g.*, CsCl concentrations of ≥0.6 mmol g_(cat)_^–1^). A detailed spectroscopic investigation illustrates how the acidity of a metal oxide surface may be modified by suitable doping.

## 2 Methods

### 2.1 Catalyst Preparation

The η-alumina reference catalyst was supplied by Ineos Chlor (Ineos Chlor catalyst ref: 25867); this is the same transition alumina examined in our previous studies^3,5−8^ and has been comprehensively characterized elsewhere.^3^ The doping of the alumina catalyst with K^+^ and Cs^+^ salts was performed by an impregnation method. In addition to the un-promoted η-alumina catalyst, five CsCl and KCl catalysts were prepared at the following dopant levels: 0.01, 0.1, 0.3, 0.6, and 1.0 mmol g_(cat)_^–1^. Details of the preparative procedures adopted and subsequent characterization of the materials are outlined elsewhere.^2,4^ The catalyst samples were activated by heating to 623 K under flowing helium (99.999%, BOC) for 150 min and then allowed to cool to ambient temperature. Throughout the experimental procedures, the sample was continuously flushed with helium gas and fed to the catalyst *via* an in-line gas purification facility (MG Oxisorb).

### 2.2 Infrared Spectroscopy and Temperature-Programed Desorption

The arrangements for undertaking the IR and temperature-programed desorption (TPD) measurements are described elsewhere.^5^ Briefly, diffuse reflectance infrared Fourier transform spectroscopy measurements were performed using an environmental chamber utilizing typically 50 mg of the catalyst. Methanol (Aldrich, 99.8+% purity) was dosed onto the catalyst at 293 K using pulse-flow techniques. All spectra are presented as background-subtracted, where a spectrum of the clean, activated catalyst has been subtracted from the dosed spectrum. No baseline or offset corrections were made. It is noted that temperatures quoted for TP-IR experiments have been corrected for a thermal gradient between the sample and the environmental chamber’s thermocouple.^9^

A detailed description of the experimental setup employed for thermal desorption experiments has been given elsewhere.^5^ Briefly, saturation of the sample could be observed by monitoring the reactor exit stream on the mass spectrometer (observing mass 31 amu corresponding to methanol). When saturation was achieved, the sample was left to purge overnight at 293 K under flowing He. TPD experiments were performed at a temperature ramp rate of 8 K min^–1^. The eluent stream from the reactor was monitored using the mass spectrometer during the TPD experiment [observing masses corresponding to methanol (31 amu), DME (45 amu), and CO (28 amu)]. Blank IR and TPD experiments performed in the absence of a catalyst sample produced unchanged baseline profiles, indicating the reported spectral changes to be solely catalyst-induced.

## 3 Results and Discussion

### 3.1 Application of IR Spectroscopy to Investigate Methanol Adsorption at 293 K on η-Alumina + CsCl and η-Alumina + KCl

**Figure 1:**
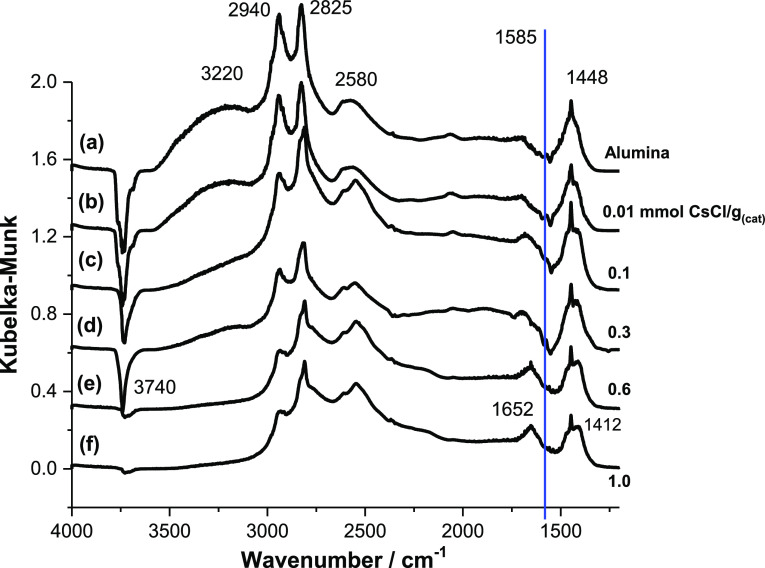
IR difference spectra for a chemisorbed overlayer of methanol adsorbed on a series of CsCl-doped η-alumina catalysts at 293 K: (a) undoped η-alumina; (b) 0.01; (c) 0.1; (d) 0.3; (e) 0.6; and (f) 1.0 mmol CsCl g_(cat)_^–1^. The blue vertical line indicates an energy of 1585 cm^–1^.

**Figure 2:**
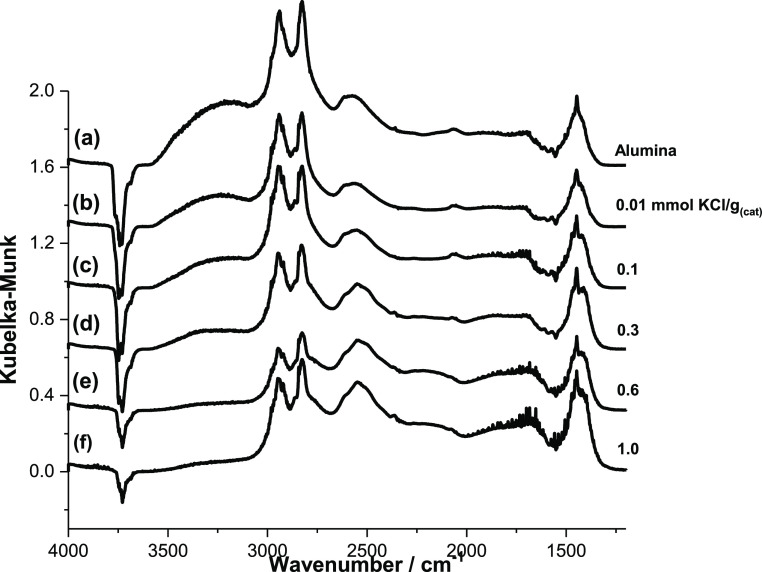
IR difference spectra for a chemisorbed overlayer of methanol adsorbed on a series of KCl-doped alumina catalysts at 293 K: (a) undoped η-alumina; (b) 0.01; (c) 0.1; (d) 0.3; (e) 0.6; and (f) 1.0 mmol KCl g_(cat)_^–1^.

Figure 1 shows the IR difference spectra obtained for a saturated overlayer of methanol at 293 K for a series of CsCl-doped η-alumina catalysts covering the range 0.01–1.0 mmol CsCl g_(cat)_^–1^, while Figure 2 shows the corresponding spectra for a series of KCl-doped catalysts at the same molar loadings of the dopant. The spectrum recorded of the base η-alumina catalyst (Figures 1a and 2a) is attributed mainly to chemisorbed methoxy species and associatively adsorbed methanol molecules and is comparable to the spectrum reported by McInroy *et al.*,^5,6^ albeit with a higher concentration of undissociated methanol.

Methyl group vibrational modes are observed at 2940 (asymmetric C–H stretch), 2825 (symmetric C–H stretch), and 1448 cm^–1^ (C–H bending mode). Increasing the concentration of the group 1 metal chloride modifier leads to a progressive decrease in the broad methanol H-bonded ν(O–H) mode located at ∼3220 cm^–1^. The associatively chemisorbed methanol is undetectable for concentrations ≥0.6 mmol CsCl g_(cat)_^–1^, leaving a spectrum characteristic of chemisorbed methoxy species.^6^

The broad feature with a bandhead at ∼2580 cm^–1^ in Figures 1a and 2a is also associated with methoxy moieties and is assigned as a combination band, with contributions from a methyl rock and methyl deformation modes.^6^ For CsCl loadings ≥0.1 mmol g_(cat)_^–1^, two components of this band are resolvable: a dominant feature at 2547 cm^–1^ and a high wavenumber shoulder at 2618 cm^–1^. The distinct bands represent a combination mode between the methyl rock and either the symmetric or asymmetric methyl deformation modes. Specifically, the former is associated with the symmetric methyl deformation [ρ(CH_3_) + δ_sym_(CH_3_)], while the latter is associated with the asymmetric methyl deformation [ρ(CH_3_) + δ_asym_(CH_3_)].^6^ The splitting of these two bands is less evident in spectra of KCl-doped catalysts.

**Figure 3:**
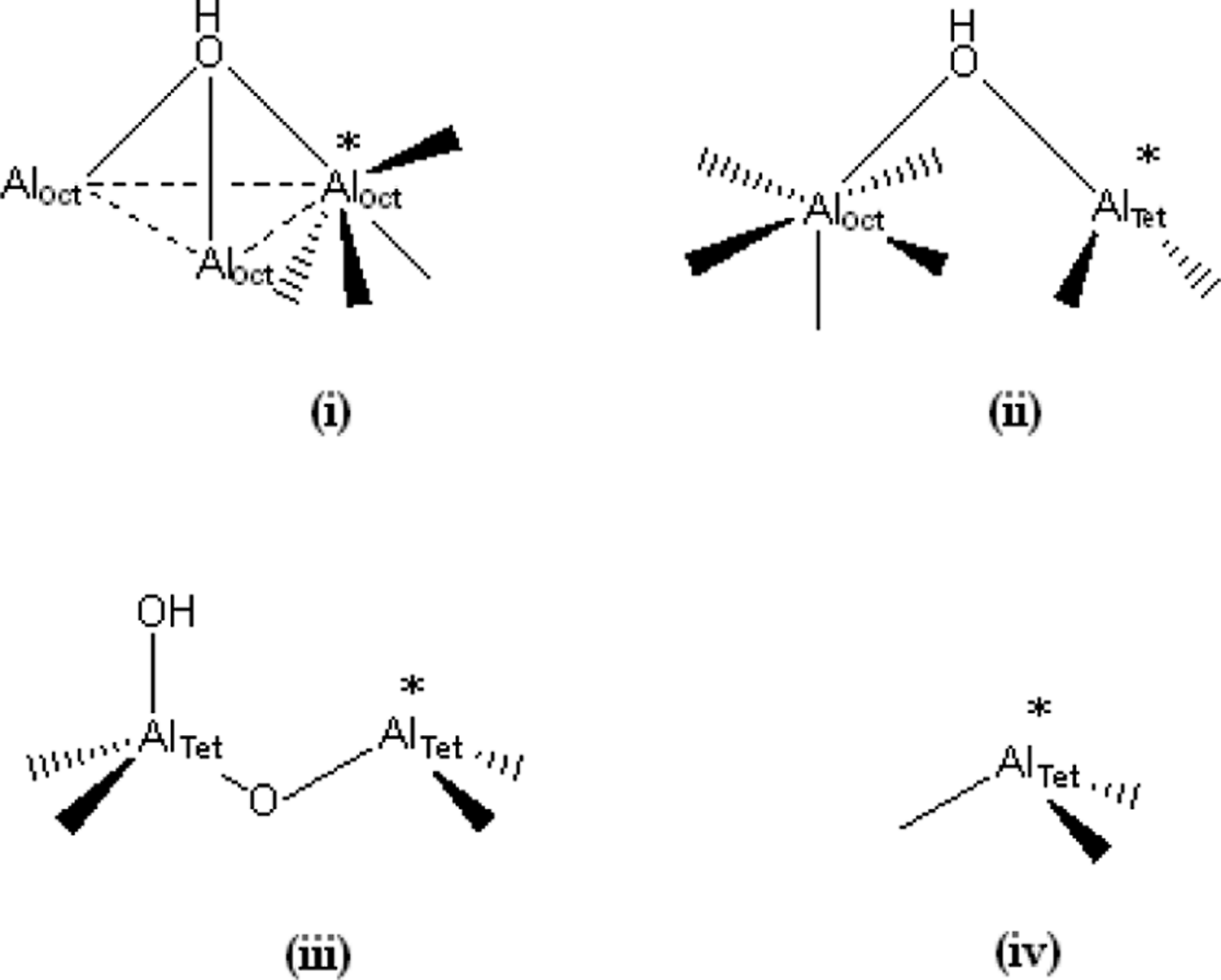
Schematic representations of the proposed acid sites of activated η-alumina: (i) weak Lewis acid site, (ii) medium-weak Lewis acid site, (iii) medium-strong Lewis acid site, and (iv) strong Lewis acid site. The asterisks indicate coordinative unsaturation.^3^ Adapted with permission from *J. Phys. Chem. B***2005**, *109*, 11592, Copyright 2005 American Chemical Society.

A prominent feature in the spectrum for η-Al_2_O_3_/CH_3_OH (Figure 1a) is a sharp negative peak at 3740 cm^–1^, which is accompanied by a shoulder to a high wavenumber at 3770 cm^–1^ and a shoulder to a low wavenumber at 3696 cm^–1^; these absorptions are assigned to hydroxyl groups adjacent to specific acidic sites on the alumina surface. Figure 3 presents a schematic diagram for the four types of active sites reported to be present on η-Al_2_O_3_.^3^ The 3770 cm^–1^ feature is assigned to terminal hydroxyl groups associated with the medium-strong Lewis acid site [moiety (iii) in Figure 3]; the intense 3740 cm^–1^ peak is assigned to hydroxyl groups associated with the medium-weak Lewis acid site [moiety (ii) in Figure 3]; and the feature at 3696 cm^–1^ is assigned to hydroxyl groups associated with the weak Lewis acid site [moiety (i) in Figure 3].^3^ On increasing the modifier concentration, the 3770 cm^–1^ feature is lost at a value of 0.1 mmol CsCl g_(cat)_^–1^. In the case of the sharp peak at 3740 cm^–1^, this feature is present over the dopant concentration range 0–0.3 mmol CsCl g_(cat)_^–1^, but intriguingly, over the dopant range 0.6–1.0 mmol CsCl g_(cat)_^–1^, the negative peak is almost completely absent, with only a weak broad feature evident with a maximum negative intensity at ∼3730 cm^–1^ but with intensity skewed to a low wavenumber down to 3677 cm^–1^ observable at 1.0 mmol CsCl g_(cat)_^–1^. These changes to the spectral profile correlate with a previous deduction from McInroy *et al.* that group 1 metal dopant levels of ≥0.6 mmol CsCl g_(cat)_^–1^ effectively neutralize the strong and medium-strong Lewis acid sites of η-alumina, leaving only medium-weak and weak Lewis acid sites accessible.^4^ The changes in the spectra of KCl-doped catalysts are similar, but the reduction in the intensity of the negative peak is less, implying a smaller effect of KCl on catalyst properties.

These spectral changes indicate changes in the hydroxyl groups associated with the acid sites, rather than the acid sites themselves, and do not convey any information about changes at the strong acid site, which lacks adjacent hydroxyl groups [Figure 3(iv)]. It should be noted that in the work on pyridine adsorption,^4^ essentially complete loss of the negative hydroxyl peaks in catalysts doped with group 1 metal chlorides was observed on adsorption of pyridine; however, the 8a mode of pyridine showed that pyridine was still bound to medium-strength sites (medium-strong and medium-weak sites are not distinguished by this mode). TPD measurements further clarified the sites of adsorption to show that only the medium-weak sites were accessible at group 1 metal loadings above 0.1 mmol CsCl g_(cat)_^–1^ and 0.3 mmol KCl g_(cat)_^–1^. It is clear, therefore, that the hydroxyl groups can be eliminated by doping with group 1 metal chlorides without blocking the corresponding acidic sites.

### 3.2 Temperature-Programed Reaction of Methanol on η-Alumina + CsCl and η-Alumina + KCl

The dehydration of methanol to produce DME over alumina surfaces (eq 2) is a well-established phenomenon.^10−12^ Matsushima and White have investigated the thermal decomposition of methanol adsorbed on alumina and identified methanol, methoxy species, formaldehyde, and DME as active surface species.^13^ In their study of methanol on η-alumina, McInroy and co-workers used temperature-programed techniques to show that heating a chemisorbed overlayer led to two primary desorption features: (i) a low-temperature methanol feature formed by methanol desorption (*via* recombinative desorption of bound methoxy groups and adsorbed hydrogen atoms) from weak and medium-weak Lewis acid sites (*T*_max_ ∼ 390 K) and (ii) a higher-temperature DME feature that arises from the reaction of methoxy species on strong and medium-strong Lewis acid sites (*T*_max_ ∼ 460 K), eq 2.^5^ Against this background, a series of post-methanol exposure TPD experiments was carried out to evaluate DME production (eq 2) from the series of CsCl- and KCl-modified η-alumina catalysts.

**Figure 4:**
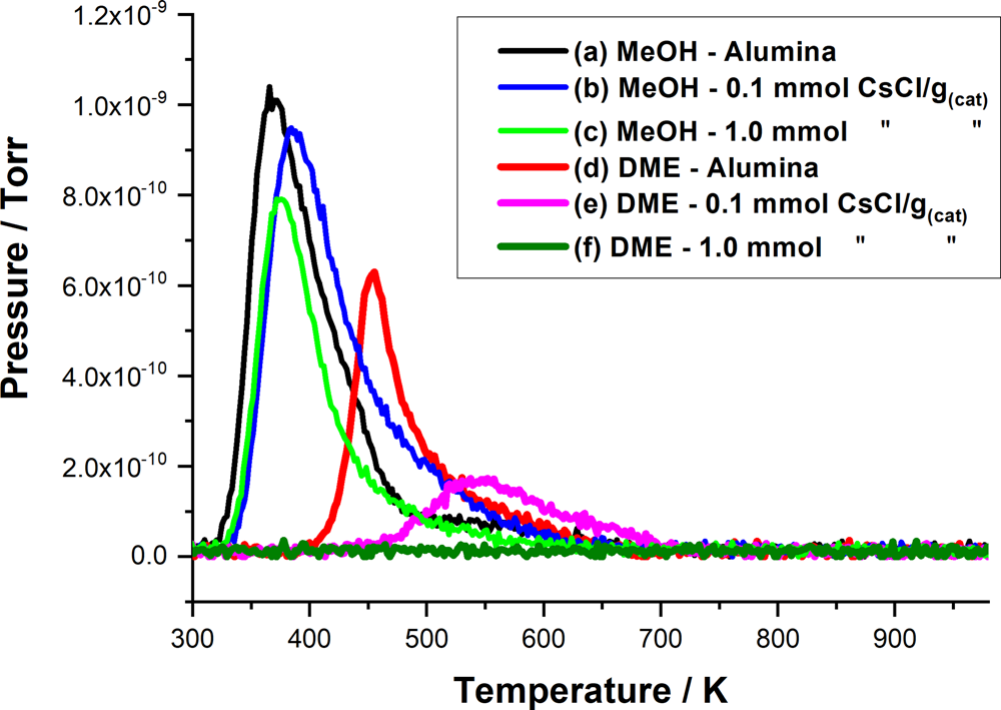
Mass selective TPD profiles obtained for methanol adsorbed on selected CsCl-doped alumina catalysts: (a) CH_3_OH (31 amu) undoped η-alumina, (b) CH_3_OH 0.1 mmol CsCl g_(cat)_^–1^, and (c) CH_3_OH 1.0 mmol CsCl g_(cat)_^–1^ and (d) CH_3_OCH_3_ (45 amu) undoped η-alumina, (e) CH_3_OCH_3_ 0.1 mmol CsCl g_(cat)_^–1^, and (f) CH_3_OCH_3_ 1.0 mmol CsCl g_(cat)_^–1^.

**Figure 5:**
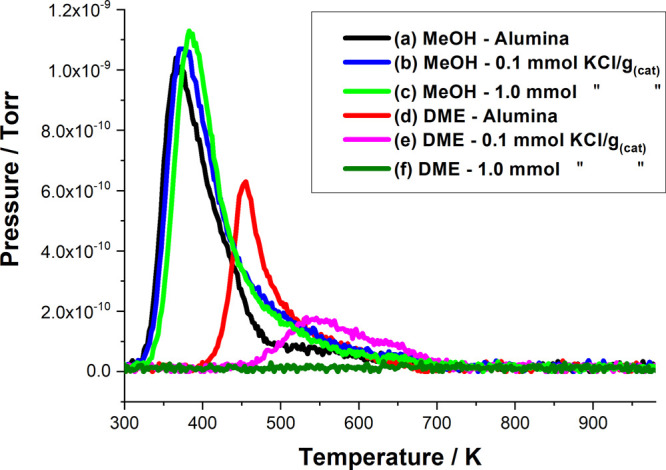
Mass selective TPD profiles obtained for methanol adsorbed on selected KCl-doped alumina catalysts: (a) CH_3_OH (31 amu) undoped η-alumina, (b) CH_3_OH 0.1 mmol KCl g_(cat)_^–1^, and (c) CH_3_OH 1.0 mmol KCl g_(cat)_^–1^ and (d) CH_3_OCH_3_ (45 amu) undoped η-alumina, (e) CH_3_OCH_3_ 0.1 mmol KCl g_(cat)_^–1^, and (f) CH_3_OCH_3_ 1.0 mmol KCl g_(cat)_^–1^.

Figures 4 and 5 show TPD profiles for methanol adsorbed on selected CsCl- and KCl-modified catalysts, respectively. In each case, profiles are reported for the standard η-alumina catalyst and the η-alumina modified *via* the addition of 0.1 and 1.0 mmol CsCl/KCl g_(cat)_^–1^. On the addition of the group 1 metal salt, both figures show a dramatic decrease in DME formation at 0.1 mmol CsCl/KCl g_(cat)_^–1^ and reduction of DME formation to below the limit of detection at 1.0 mmol CsCl/KCl g_(cat)_^–1^.

**Figure 6:**
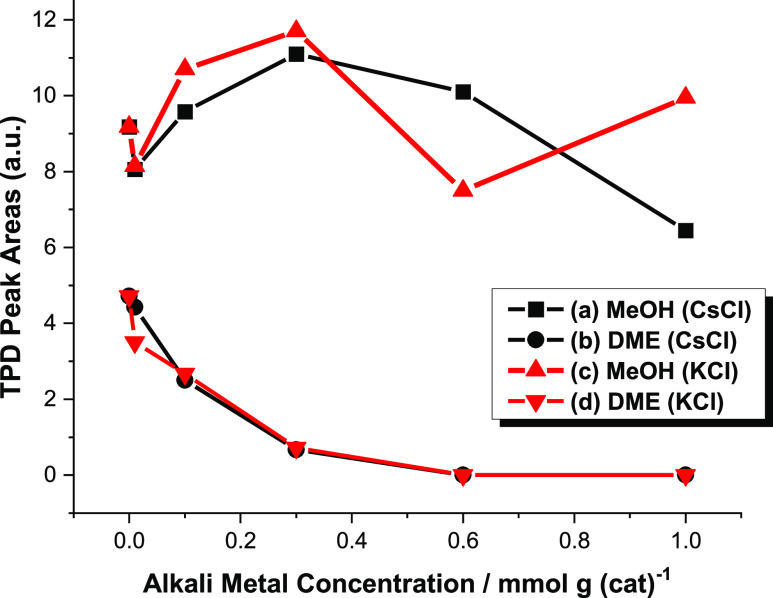
TPD areas for MeOH and DME formation over the range of CsCl- and KCl-doped η-alumina catalysts (Figures 4 and 5): (a) CsCl, CH_3_OH; (b) CsCl, CH_3_OCH_3_; (c) KCl, CH_3_OH; and (d) KCl, CH_3_OCH_3_.

Figure 6 presents MeOH and DME TPD peak areas as a function of group 1 chloride loading and shows that the addition of the chemical modifier has a much greater effect on DME formation/desorption than methanol desorption. Although some variability is observed in the integrated methanol peak area as a function of temperature, particularly for CsCl (Figure 4), a reduction in peak area over the temperature range studied does not exceed 20% over the coverage range studied. In dramatic contrast, higher loadings reduce the DME feature to below the detection limit. Specifically, at dopant loadings of 0.1 mmol g_(cat)_^–1^, medium-strong Lewis acid sites are still available on the η-alumina surface,^4^ and reduced levels of DME formation are observed. However, at the highest dopant concentration examined, all strong and medium-strong Lewis acid sites have been selectively neutralized,^4^ and, importantly, DME formation is reduced below the detection limit.

Collectively, these methanol TPD results complement the earlier pyridine studies^4^ and reaffirm the concept of site-selective chemistry, with group 1 metal salts selectively neutralizing Lewis acid sites associated with the formation of the unwanted byproduct (DME) in the methyl chloride synthesis process (eq 2). It is noteworthy that despite the removal of physisorbed methanol and undissociated methanol and the blocking of strong and medium-strong acid sites, the quantity of methanol adsorbed and desorbed as unchanged methanol remains largely unaffected, showing the presence of other sites for adsorption; this stands in stark contrast to the results with pyridine, which show an almost 10-fold reduction in the desorbed pyridine between undoped alumina and alumina doped with 1 mmol CsCl g_(cat)_^–1^.^4^

### 3.3 Temperature-Programed IR Spectroscopy and Temperature-Programed Desorption of Methanol on η-Alumina + CsCl and η-Alumina + KCl

In addition to the absorptions due to adsorbed methanol, Figures 1 and 2 show additional spectral features at those CsCl and KCl concentrations that lead to the dramatic attenuation intensity of the alumina negative ν(O–H) bands at about 3740 cm^–1^; these additional spectral features are assigned to formate (see below). In their study of methanol on η-alumina, McInroy and co-workers used temperature-programed techniques and found that temperatures above 573 K led to the formation of a symmetrically bound formate species which, on further warming to ≥673 K, decomposed as evidenced by the desorption of CO and H_2_O.^5^ The presence in Figure 1 of formate was unexpected under such mild conditions (293 K), so a program of temperature-programed IR spectroscopy and desorption studies was undertaken to investigate the formation and decomposition of the formate entities.

The additional spectral features are different from those previously observed for formate and arise at lower temperatures. The assignment is justified by consideration of the absorption frequencies observed on surfaces by other investigators and by elimination of the other possible candidates. In the spectra taken at higher group 1 metal chloride loadings, two additional changes to the spectra are observed, *viz.*, a band at 1652 cm^–1^ becomes prominent, and a broad shoulder at 1412 cm^–1^ is observed which is resolvable from the methoxy δ(CH_3_) mode at 1451 cm^–1^. An inflection at about 2774 cm^–1^ is also observed on the ν_(s)_(CH_3_) peak observed at 2825 cm^–1^. In principle, a band at about 1640 cm^–1^ could be attributable to the δ(OH) mode of adsorbed water molecules, but the absence of a ν(OH) feature in Figure 1e,f excludes that possibility. Alternatively, the peaks at 1652 and 1412 cm^–1^ could represent, respectively, the ν_asym_(OCO) and ν_sym_(OCO) modes of bicarbonate entities.^14^ However, this possibility is excluded due to the absence of an associated ν(OH) mode at about 3620 cm^–1^.^14^ Moreover, bicarbonate formation would require a source of CO_2_,^15^ which is inaccessible to the reaction system. Similarly, a role for carbonate ions is rejected; the reduction of carbonate to formate is thought to be implausible, and there is no evidence for carbonate formation in the spectra for undoped η-alumina (Figure 1a). Instead, and rather surprisingly, the spectral changes observed in Figure 1 are believed to signify that on methanol dosing, CsCl concentrations of ≥0.6 mmol g_(cat)_^–1^ induce the formation of formate species at room temperature which reside alongside a population of methoxy species. Specifically, the 1652 and 1412 cm^–1^ bands represent, respectively, the ν_asym_(COO) and δ(CH) modes of formate species. The weak feature at 2774 cm^–1^ is assigned to the formate ν(C–H) mode.

Comparable peaks for chemisorbed formate on potassium-modified Ru(001) are reported by Weisel and co-workers.^16^ Using the technique of reflection absorption infrared spectroscopy, the vibrational spectrum for formate species adsorbed on a multilayer of potassium deposited on a Ru(001) crystal shows formate ν(C–H), ν_asym_(COO), and δ(C–H) modes to be present at, respectively, 2780, 1649, and 1385 cm^–1^. The IR spectrum of crystalline potassium formate shows the ν_asym_(COO) mode to be present at 1585 cm^–1^; therefore, the adsorbed formate feature represents a significant hypsochromic shift of 64 cm^–1^, which is attributed to a reduced symmetry compared to the expected *C*_2*v*_ point group.^16^ With reference to Figure 1f, the peak at 1652 cm^–1^ corresponds to a hypsochromic shift of 67 cm^–1^ (the blue line in Figure 1 indicates 1585 cm^–1^). This band is broader than that observed for formate chemisorption on η-alumina,^5^ consistent with adoption of a less symmetric environment in the presence of high coverages of the group 1 metal salt, possibly *C*_*s*_ or *C*_1_.^16^Table 1 presents the band assignments for Figure 1.

**Table 1:** Vibrational Assignment of Infrared Bands Observed for a Saturation Exposure of Methanol on η-Alumina at 298 K: (a) Unmodified η-Alumina; (b) Components of the RT Spectrum Attributed to Methoxy Species; and (c) Assignment of Additional Bands in the Room-Temperature Methanol Spectrum at CsCl Loadings of ≥0.6 mmol g_(cat)_^–1^

assignment	(a) η-alumina/CH_3_OH (cm^–1^)^a^	(b) adsorbed methoxy species (cm^–1^)^b^	(c) adsorbed formate species observed in the RT spectrum at elevated CsCl loadings (cm^–1^)^c^
ν_sym_(CO_2_^–^)			
δ(CH)			1412
δ(CH_3_)	1448	1448	
ν_asym_(CO_2_^–^)			1652
ρ(CH_3_) + δ_sym_(CH_3_)		2547	
ρ(CH_3_) + δ_asym_(CH_3_)		2618	
ν(CH)			2774
ν_sym_(CH_3_)	2825	2825	
ν_asym_(CH_3_)	2940	2940	
ν(OH)_H-bonded_	3220		
ν(OH)_weak_	3696		
ν(OH)_medium-weak_	3742		
ν(OH)_medium-strong_	3770		

a Figure 1a.

b Figure 1e.

c Figure 1f.

The presence of formate at 293 K for CsCl and KCl concentrations ≥0.6 mmol g_(cat)_^–1^ hints at an additional chemical effect, over and above that contained within the Lewis acid site neutralization model.^4^ To investigate how the doped η-Al_2_O_3_/CH_3_O_(ad)_/HCO_2(ad)_ surfaces, as evidenced in Figures 1 and 2, responded to increasing temperature, temperature-programed measurements of the methanol-dosed and group 1 chloride-doped η-alumina samples were undertaken. First, this involved IR measurements recorded after thermal ramping to temperatures associated with inducing formate formation (653 K) and decomposition (743 K).^5^ These were then followed up by mass spectrometric measurements of CO desorption that provides additional information on the decomposition process.^5^

**Figure 7:**
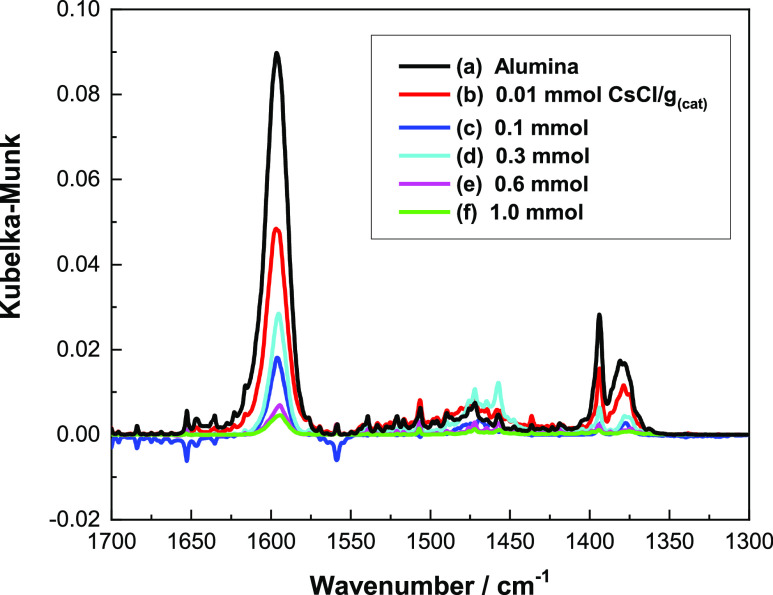
IR spectra for a saturated overlayer of methanol adsorbed on a range of CsCl-doped catalysts and heated to 653 K: (a) undoped η-alumina; (b) 0.01; (c) 0.1; (d) 0.3; (e) 0.6; and (f) 1.0 mmol CsCl g_(cat)_^–1^.

Figure 7 shows the IR difference spectra for a saturated overlayer of methanol adsorbed at room temperature on a range of CsCl-doped alumina catalysts that are heated to 653 K, a temperature above that at which the methoxy → formate transition on η-alumina^5^ initiates. The spectra are shown in the region 1700–1300 cm^–1^ and uniquely correspond to the presence of formate species.

Figure 7a shows the spectrum for a saturated overlayer of methanol over the standard (undoped) η-alumina catalyst that has been heated from 298 to 653 K to exhibit a series of bands at 1596, 1393, and 1378 cm^–1^, which are, respectively, assigned to the antisymmetric CO_2_ stretch, the C–H bend, and the symmetric CO_2_ stretch of surface formate.^5^ The sharp and symmetric band at 1596 cm^–1^ is indicative of formate species residing in a discrete adsorption site,^5^ most probably exhibiting *C*_2*v*_ symmetry.^16^ Importantly, the broad band observed at 1652 cm^–1^ in Figure 1e,f is absent in Figure 7, thereby providing a distinction for two types of surface formate species. First, methanol saturation at room temperature on alumina containing high CsCl loadings leads to a perturbed formate species, with the broadness of the ν_asym_(COO) mode in Figure 1e,f suggestive of the formate occupying a range of adsorption sites. Second, the formate spectrum observed in Figure 7a shows that the ambient-temperature formate species evidenced in Figure 1 differs from the thermally activated methoxy → formate transformation over η-alumina previously described by McInroy and co-workers.^5^ The absence of those formate absorptions observed in Figure 1e,f from Figure 7 for all CsCl coverages studied indicates this species to have decomposed, desorbed, or migrated to the high-symmetry site on warming to 653 K.

It is also noteworthy that the nature of the formate species formed *via* the thermally activated pathway is identical at all the CsCl loadings examined in this work. By comparison with Figure 4, Figure 7 shows that formate formation is taking place at temperatures above those where methanol desorption is complete, and DME desorption is almost complete. It is clear, therefore, that some methanol is being bound tightly to the surface but in a site that does not favor DME formation.

It is interesting that the intensity of the absorptions due to formate appears to follow a trend, with most formate present at zero doping and least formate present at the highest group 1 metal salt loading; however, the intensity of absorption at 0.3 mmol CsCl g_(cat)_^–1^ seems anomalous as it is higher than that at 0.1 mmol g_(cat)_^–1^, and so the complexity of the formate was further investigated by obtaining the IR spectra for a methanol overlayer that has been heated to 743 K, a temperature that exposes the formate decomposition regime.^5^

**Figure 8:**
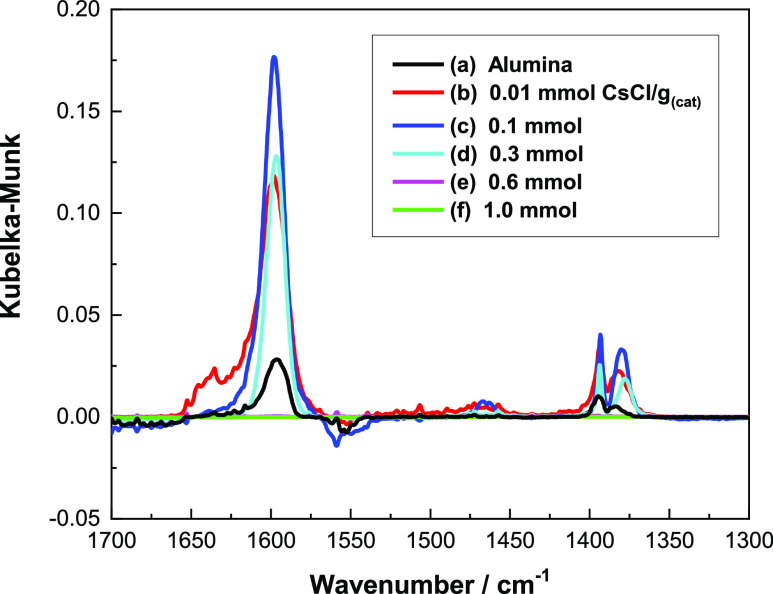
IR spectra for a saturated overlayer of methanol adsorbed on a range of CsCl-doped catalysts and heated to 743 K: (a) undoped η-alumina; (b) 0.01; (c) 0.1; (d) 0.3; (e) 0.6; and (f) 1.0 mmol CsCl g_(cat)_^–1^.

Figure 8 presents the IR spectra for a room-temperature-dosed chemisorbed overlayer of methanol on CsCl-doped η-alumina after heating to 743 K. The intensity of the antisymmetric CO_2_ stretch at 1598 cm^–1^ shows that nearly complete formate decomposition is observed over un-doped η-alumina at this temperature.^5^Figure 8a shows the undoped η-alumina to exhibit minimal peak intensity, indicating thermally induced formate decomposition to be a facile process at 743 K. This observation reproduces results reported by McInroy *et al.*(5)Figure 8b,c shows the intensity of this mode to increase over the range 0.01–0.1 mmol CsCl g_(cat)_^–1^; however, peak intensity decreases in Figure 8d (0.3 mmol CsCl g_(cat)_^–1^), and no formate is detected in Figure 8e,f. Interestingly, Figure 8b uniquely shows the additional presence of a feature at 1635 cm^–1^, a frequency representative of the unsymmetric formate species under consideration in Section 3.3.

**Figure 9:**
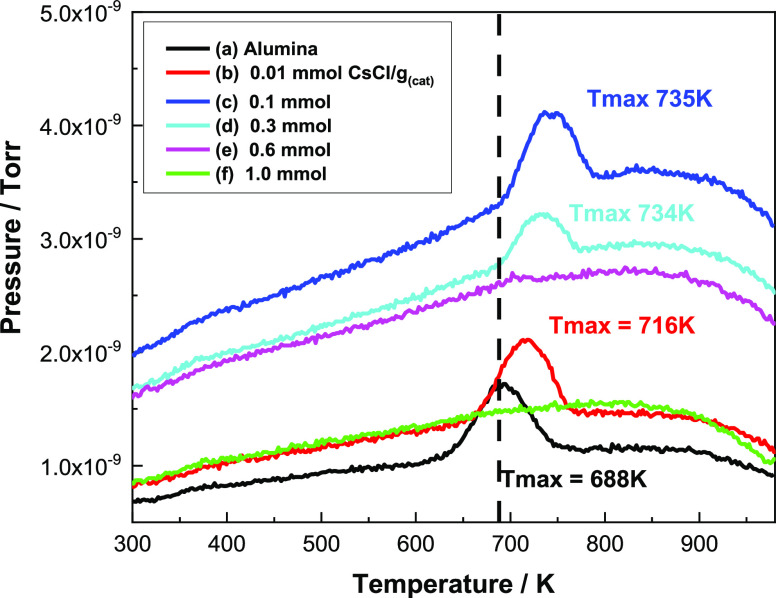
Mass spectrometry profiles obtained for CO evolution (28 amu) from methanol TPD experiments performed on a range of CsCl-doped catalysts: (a) undoped η-alumina; (b) 0.01; (c) 0.1; (d) 0.3; (e) 0.6; and (f) 1.0 mmol CsCl g_(cat)_^–1^.

To investigate these trends, TPD measurements for chemisorbed methanol were undertaken, concentrating on CO evolution as an indicator of the formate decomposition process.^5^Figure 9 shows the resulting profiles. In agreement with results reported previously with stepwise heating,^5^ the un-doped η-alumina (black line) exhibits a single broad feature with a *T*_max_ of 688 K. The temperature of the peak maximum increases for CsCl-doped catalysts. There is a complete absence of a CO feature in the profiles for loadings ≥0.6 mmol CsCl g_(cat)_^–1^, which possibly reveals a reduced sensitivity compared with IR spectroscopy, as formate is discernible in Figure 7e,f.

**Figure 10:**
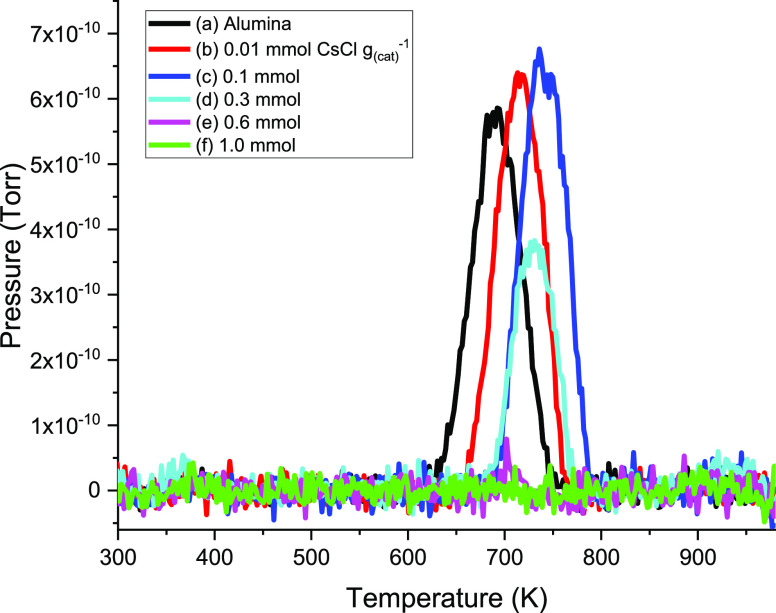
Background-subtracted CO evolution from TPO profiles presented in Figure 9: (a) undoped η-alumina; (b) 0.01; (c) 0.1; (d) 0.3; (e) 0.6; and (f) 1.0 mmol CsCl g_(cat)_^–1^.

There are several points to note from Figure 9. First, despite there being measurable quantities of formate produced at room temperature on catalysts with high loadings of CsCl (Figure 1), Figure 9 shows no peak corresponding to the desorption or decomposition of this formate; possibly this indicates a limit to the sensitivity of the TPD measurements or that decomposition follows a different path. Second, there is a pronounced peak for CO emission in catalysts with 0–0.3 mmol CsCl g_(cat)_^–1^ and no peak for catalysts with ≥0.6 mmol g_(cat)_^–1^; it is therefore a reasonable deduction that the peaks observed in Figure 9 correspond to the decomposition to carbon monoxide of the formate observed by IR (Figures 7 and 8). Figure 9 is complicated by sloping baselines. Therefore, to more clearly define the formate decomposition process, Figure 10 presents the baseline-corrected TPD profiles for the 0–0.3 mmol CsCl g_(cat)_^–1^ data set.

Figure 10 reveals that the onset and maxima of CO emissions are taking place at different temperatures, despite the peak positions of the IR spectra of the formate on all the catalyst samples being essentially identical (Figure 7)—such decomposition temperatures are therefore not likely related to binding energies, which would affect the frequencies of absorption. Similar peak maxima trends are also observed when using η-alumina catalysts modified by the addition of KCl. Table 2 shows the peak maxima recorded for CO evolution for the range of CsCl- and KCl-doped catalysts. It is noted that on comparing the effects of the different group 1 modifiers, only marginal differences in *T*_max_ are encountered. Repeat measurements were not undertaken; therefore, the degree of variance is unknown. Nonetheless, Table 2 shows that CsCl consistently conveys a higher *T*_max_ for CO desorption.

**Table 2:** Peak Maxima Recorded for CO Evolution Profiles Obtained during Methanol Temperature-Programed Desorption Experiments

CsCl-doped catalysts/mmol g_(cat)_^–1^	*T*_max_ CO/K	KCl-doped catalysts/mmol g_(cat)_^–1^	*T*_max_ CO/K
0	688	0	688
0.01	716	0.01	706
0.1	740	0.1	726
0.3	730	0.3	728
0.6		0.6	
1.0		1.0	

**Figure 11:**
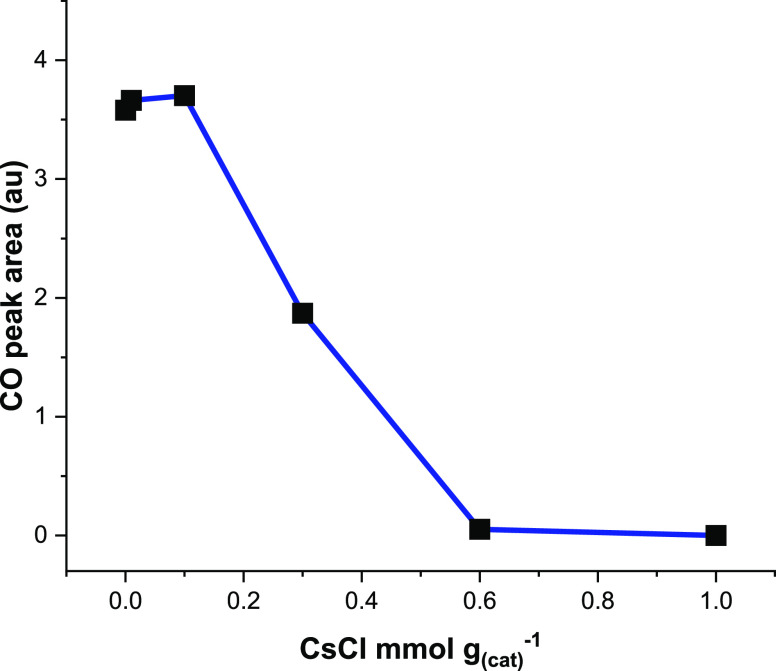
Integrated CO intensity from the background-subtracted TPO profile (Figure 10) as a function of the CsCl concentration.

Figure 11 presents the area of the background-subtracted CO TPD profiles (Figure 10) with respect to CsCl loading and shows the quantity of CO desorption to be approximately constant over the range 0–0.1 mmol CsCl g_(cat)_^–1^. Within the context of the “acid site neutralization/titration model”,^4^ it is suggested that Figure 11 indicates formate formation to be associated with the medium-weak Lewis acid site. It was shown that the medium-strong Lewis acid sites were effectively capped at group 1 metal chloride loadings of 0.1 mmol CsCl g_(cat)_^–1^, whereas the medium-weak acid site concentration was progressively decreased at 0.3 mmol CsCl g_(cat)_^–1^ and above, a trend followed by the CO peak areas shown in Figure 11. Furthermore, the anomalous intensity profile of the antisymmetric CO_2_ stretch evident in Figure 8 is thought to reflect that the dopant is simultaneously perturbing the rates of formate formation and decomposition. More work is necessary to better understand this latter topic.

Unexpectedly, the ambient-temperature IR spectra for methanol over CsCl- and KCl-doped η-alumina show evidence for formate formation at dopant levels ≥0.6 mmol CsCl or KCl g_(cat)_^–1^, which concomitantly correspond to attenuation of negative alumina ν(O–H) bands at 3770 and 3740 cm^–1^ which are, respectively, attributed to medium-strong and medium-weak Lewis acid sites.^3^Figure 1 indicates that at higher group 1 chloride coverages, the surface chemistry is perturbed beyond that of a simple Lewis acid site neutralization model. Group 1 metal salts are known to convey Brønsted basicity to metal oxide surfaces, enabling base-catalyzed reactions such as Michael additions to be facilitated.^17^ Thus, at the higher group 1 doping levels (≥0.6 mmol g_(cat)_^–1^), it is proposed that new Brønsted base sites are formed. The ambient-temperature IR spectra (Figures 1 and 2) show no evidence for the addition of H_2_O or OH^–^ species over the ≥0.6 mmol CsCl g_(cat)_^–1^ coverage range, suggesting that a candidate for the induction of Brønsted basicity is the anhydrous chloride ion. The tentative suggestion is that a surface Al–OH group neighboring a Lewis acid site is deprotonated by Cl^–^ to form HCl.

**Figure 12:**
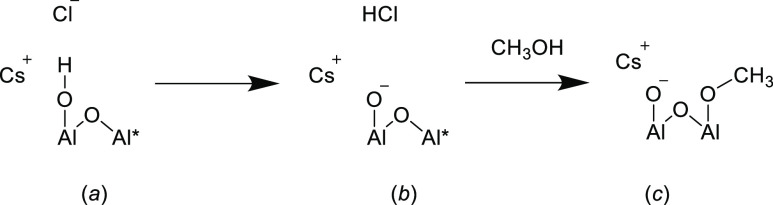
Reaction scheme to illustrate how high CsCl loadings induce Brønsted basicity at the η-alumina surface and how the resulting surface interacts with methanol at room temperature. (a) CsCl reacts with a hydroxyl group; (b) this leads to formation of a Brønsted base site and the co-production of HCl; (c) subsequent exposure to methanol leads to methoxy formation at a Lewis acid site, as signified by an asterisk (*).

It is informative to consider the significance of the threshold dopant concentration necessary to induce the ambient-temperature formate formation. Figure 1 shows evidence of formate formation for CsCl loadings of ≥0.6 mmol g_(cat)_^–1^. The Brunauer–Emmett–Teller surface area for η-Al_2_O_3_ + 0.6 mmol CsCl g_(cat)_^–1^ is 186 m^2^ g^–1^.^4^ Assuming reasonable values for ionic radii,^18^ this loading equates to a CsCl surface area of 137 m^2^ g^–1^, corresponding to a surface coverage, θ, of ∼0.74, that is, close to monolayer coverage. As described in Section 2.2, the IR spectra are presented as difference spectra. Thus, the negative alumina ν(O–H) features at about 3740 cm^–1^ evident in the spectrum of the undoped η-alumina (Figure 1a) indicate hydrogen bonding of these entities in the methanol chemisorption process.^5^ However, the dramatic attenuation of the negative alumina ν(O–H) features at ≥0.6 mmol CsCl g_(cat)_^–1^ (Figure 1e) indicates this threshold CsCl coverage to be causing the consumption of surface hydroxyl groups so that upon methanol exposure, negligible negative peaks are observed in the resulting difference spectrum. Figure 12 presents a proposed reaction scheme for how CsCl induces Brønsted basicity and modifies the methanol chemisorption process. Figure 12a considers the addition of CsCl to η-alumina; the asterisk signifies a Lewis acid site. The chloride ion induces deprotonation of a hydroxyl group and formation of HCl, whereas the cation stabilizes the resulting negative charge on the oxygen atom. This step [process (a) → (b)] is thought to be entropically favored. Methanol adsorption occurs at the Lewis acid site, leaving the oxygen anion undisturbed. Upon linking Figure 12 to 1, subtraction of the IR background spectrum of the CsCl-doped catalyst (Figure 12b) from the spectrum of chemisorbed methanol over CsCl-doped η-alumina (Figure 12c) would lead to no significant negative deflections in the hydroxyl stretching region, as is observed experimentally.

It is acknowledged that the HCl indicated in Figure 12 is unassigned, that is, it is unspecified with respect to adsorption or as a gaseous product. Previous work has established that HCl reacts with η-Al_2_O_3_*via* a dissociative adsorption process.^7^ Therefore, it is possible that HCl could be present on regions of bare η-Al_2_O_3_, but the IR spectra of Figures 1 and 2 show no evidence for HCl adsorption. Alternatively, the group 1 metal chloride concentration could be sufficient to minimalize the adsorption processes so that gaseous HCl forms and is vacated from the system by the He carrier gas. In either case, the quantities of HCl produced *via* the process depicted in Figure 12 would be small, making detection of HCl by IR or mass spectrometry difficult. As no evidence for either adsorbed or gaseous HCl is evident from the studies undertaken, the fate of HCl cannot be specified.

Although the CsCl- and KCl-doped η-alumina/methanol ambient-temperature IR spectra (Section 3.1) are broadly comparable, it is evident that the spectral profile is more changed in the presence of the Cs salt compared with the K salt. Most noticeably, the degree of attenuation of the negative-intensity alumina ν(O–H) modes at about 3740 cm^–1^ is relatively reduced on doping with KCl (Figure 2) compared to that observed when CsCl is the dopant (Figure 1). It is suggested that KCl is less reactive than CsCl with the alumina hydroxyl group as indicated in Figure 12a so that at comparable dopant concentrations, a population of surface hydroxyl groups remains to react with adsorbed methanol. This outcome is also consistent with the relatively lower concentrations of formate on the KCl-doped catalyst observed in Figure 2. In this way, the cation of the group 1 chloride maintains an effect on the surface environment. It is possible that the relative surface mobilities of the two salts could be influencing the surface chemistry.

The observations made indicate that adsorbed formate species may be formed on η-alumina from chemisorbed methoxy species either by (i) conventional thermally induced activation (Section 3.3)^5,10^ or (ii) at ambient temperatures upon doping with relatively high concentrations of group 1 metal chlorides (Section 3.1). These observations may be rationalized according to the following three stages using CsCl as a representative dopant material: (I) Under initial catalyst activation conditions, CsCl creates Brønsted base sites as illustrated in Figure 12a which are responsible for the room-temperature formate species observed in Figures 1 and 2. (II) At high CsCl loadings, thermally induced formate is not made, but at intermediate concentrations, formate is produced at a lower rate than on undoped alumina (Figure 7). (III) At still higher temperatures (743 K), formate undergoes surface-catalyzed decomposition. This is rapid on undoped alumina but slower on intermediate doped aluminas. Formate formation does not occur ≥0.6 mmol CsCl g_(cat)_^–1^ (Figures 7 and 8 and Table 2).

These refinements for how methanol binds to a modified η-Al_2_O_3_ sample are informative, and it is useful to reflect on how the methanol-derived entities may combine with HCl over this surface. McInroy and co-workers have reported on methyl chloride selectivity enhancements attainable on CsCl-/KCl-modified η-Al_2_O_3_ and proposed a scheme for how methoxy species and chlorine, formed *via* the dissociative adsorption of methanol and HCl, respectively, are adsorbed at adjacent medium-weak Lewis acid sites, which then combine to selectively form methyl chloride.^2^ The subtleties observed in the present work concerning variations in adsorbed formate geometry are thought to not cause any deviation from the previously proposed reaction model.

**Figure 13:**
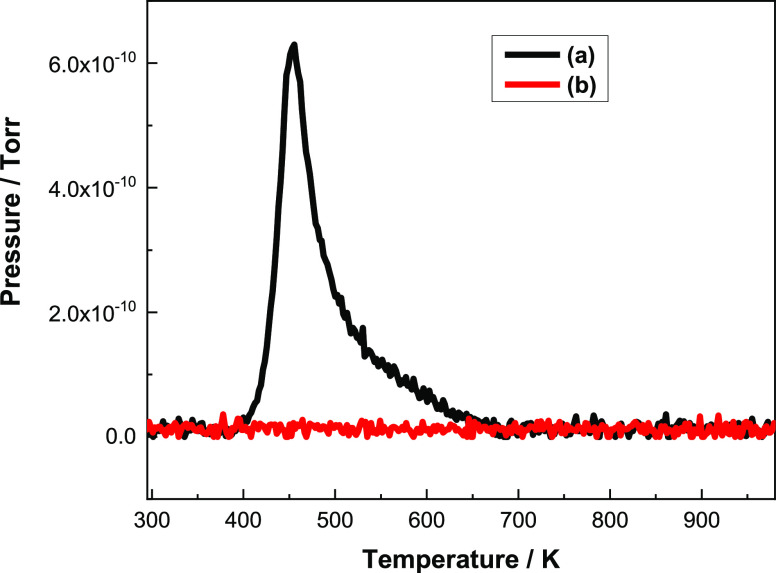
TPD profiles for DME evolution (45 amu) for two alumina samples exposed to a saturation coverage of methanol at 293 K: (a) undoped η-alumina (black line) and (b) η-alumina + 1.0 mmol CsCl g_(cat)_^–1^ (red line).

Finally, as indicated in the Introduction, minimization of eq 2 is associated with a successful methyl chloride synthesis catalyst. As an illustration of how a group 1 metal chloride can be used to modify by-product formation over the base η-alumina catalyst, Figure 13 presents the TPD profiles for DME evolution (45 amu) from two alumina samples exposed to a saturation coverage of methanol at 298 K: (a) undoped η-alumina and (b) η-alumina + 1.0 mmol CsCl g_(cat)_^–1^. First, as evidenced elsewhere,^5^Figure 13a shows that the non-modified η-alumina yields a distinct DME feature that exhibits a *T*_max_ value of 448 K that is skewed to high temperature. In dramatic contrast, Figure 13b shows that for the η-alumina + 1.0 mmol CsCl g_(cat)_^–1^ sample, no DME is detected. Figure 13 vividly demonstrates how this modifier applied at this loading suppresses DME formation below the detection limit as η-alumina/methanol is linearly temperature-ramped from ambient to 1000 K. Thus, although the surface chemistry observed is seen to exhibit complexity, as outlined above, this work further justifies and endorses the use of specific loadings of group 1 metal chlorides to minimize DME formation over an η-alumina catalyst. These outcomes provide baseline understanding for the optimization of methyl chloride synthesis catalysis, as outlined in the patent literature.^19^

## 4 Conclusions

This work has used a combination of methanol chemisorption coupled with IR spectroscopy and TPD measurements to examine how the addition of group 1 metal chlorides over the concentration range of 0–1.0 mmol g_(cat)_^–1^ modifies the acid site distribution of an η-alumina catalyst. At room temperature, methoxy species dominate and co-exist alongside formate species in a low-symmetry environment [ν_asym_(COO) = 1652 cm^–1^]. Warming the sample leads to the loss of the latter species and the formation of formate in a high-symmetry environment [ν_asym_(COO) = 1596 cm^–1^], whereas TPD studies show methanol desorption to be only marginally affected on increasing the modifier concentration, and at higher coverages, DME formation is reduced to below the limit of detection. This outcome forms the basis of an industrial specification catalyst.^19^ In addition to increasing modifier concentrations progressively and decreasing the accessibility of Lewis acid sites, the introduction of Brønsted basicity at the catalyst surface is additionally induced.
